# Immunologic derangement caused by intestinal dysbiosis and stress is the intrinsic basis of reactive arthritis

**DOI:** 10.1007/s00393-024-01480-4

**Published:** 2024-02-25

**Authors:** Tao He, Weiqing Qian

**Affiliations:** 1 Nanjing University of Chinese Medicine, Nanjing, China; 2 Nanjing City Hospital of Chinese Medicine, 157, Daming Road, Nanjing, Qinhuai District China

**Keywords:** Intestinal dysbiosis, Stress, Immunologic derangement, Reactive arthritis, Autoimmune disease, Darmdysbiose, Stress, Immunologische Störung, Reaktive Arthritis, Autoimmunerkrankung

## Abstract

Reactive arthritis (ReA) is defined as arthritis resulting from infections in other body parts, such as the gastrointestinal and urogenital tracts. The primary clinical manifestations involve acute-onset and self-limiting asymmetric large joint inflammation in the lower limbs. Although bacterial or chlamydia infections have long been recognized as playing a pivotal role in its pathogenesis, recent studies suggest that antibiotic treatment may perpetuate rather than eradicate chlamydia within the host, indicating an involvement of other mechanisms in Reactive arthritis. Reactive arthritis is currently believed to be associated with infection, genetic marker (HLA-B27), and immunologic derangement. As an autoimmune disease, increasing attention has been given to understanding the role of the immune system in Reactive arthritis. This review focuses on elucidating how the immune system mediates reactive arthritis and explores the roles of intestinal dysbiosis-induced immune disorders and stress-related factors in autoimmune diseases, providing novel insights into understanding reactive arthritis.

## Abstract (de)

Reaktive Arthritis (ReA) ist definiert als Arthritis, die das Resultat von Infektionen in anderen Körperteilen darstellt, z. B. im Gastrointestinal- und Urogenitaltrakt. Zu den primären klinischen Manifestationen gehören selbstlimitierende asymmetrische Entzündungen großer Gelenke in den unteren Extremitäten mit akutem Beginn. Zwar galten bakterielle oder Chlamydieninfektionen lange als entscheidend in der Pathogenese, aber aktuellen Studien zufolge führt eine antibiotische Behandlung möglicherweise eher zur Aufrechterhaltung als zur Eradikation der Chlamydieninfektion im Wirt, was auf die Beteiligung anderer Mechanismen an der Reactive arthritis hinweist. Derzeit wird davon ausgegangen, dass Reactive arthritis mit Infektionsprozessen, einem genetischen Marker (HLA-B27) und immunologischen Störungen einhergeht. Als Autoimmunerkrankung ist dem Verständnis der Rolle des Immunsystems bei Reactive arthritis zunehmend Aufmerksamkeit gewidmet worden. Der Fokus der vorliegenden Arbeit liegt auf der Erläuterung der Mediationsvorgänge des Immunsystems bei Reactive arthritis und auf der Darstellung der Bedeutung von durch intestinale Dysbiose induzierte Immunkrankheiten und stressbedingte Faktoren bei Autoimmunerkrankungen, was neue Einsichten in das Verständnis der Reactive arthritis vermittelt.

## Introduction

Arthritis patients are frequently encountered in orthopedic clinics; however, it should be noted that there exists a type of arthritis that is not confined to local joint lesions but rather represents a group of systemic diseases known as spondylarthritis. Spondylarthritis encompasses various interconnected multisystem inflammatory conditions primarily characterized by joint damage along with involvement of surrounding tissues. Reactive arthritis (ReA) is one form within this spectrum.

Reactive arthritis, formerly known as Reiter’s syndrome, refers to an autoimmune disease marked by noninfectious joint inflammation triggered by an extra-articular infection typically originating from either the urogenital or gastrointestinal tract, which can involve multiple systems throughout the body [1]. The onset of ReA typically occurs 1–3 weeks following infection and may present acutely or insidiously [2]. It usually affects individuals between the ages of 20 and 40 [3], with adults being more susceptible than children, and the proportion of affected men and women varying in different circumstances [1, 4]. Due to differences in clinical symptoms, test methods, diagnostic criteria, and geographical environment, epidemiological studies on reactive arthritis are challenging [5, 6]. Incidence, prevalence, and attack rates vary widely among different studies [4].

Clinical manifestations range from mild local conditions to severe systemic multisystem diseases that can be divided into joint symptoms and extraarticular multisystem manifestations. The typical manifestation is asymmetrical oligoarthritis of lower limb joints such as the knee joint, ankle joint, hip arthritis, or toe arthritis [7]. ReA presents as immune-mediated synovitis [8, 9], leading to joint pain, swelling, and dysfunction. Extra-articular manifestations include enthesitis, tendinitis, and bursitis. Skin mucosal lesions, such as blisters on the palms and soles of the feet, may occur and some patients will have oral ulcers. Gastrointestinal symptoms, urinary symptoms, and conjunctivitis symptoms may still occur. Patients may be accompanied by fever, fatigue, muscle aches, loss of appetite, and other general discomfort symptoms [10]. Furthermore, about one third of patients experience low back pain [11] and cardiac involvement is frequently observed [12].

## Etiology of reactive arthritis

Unlike rheumatoid arthritis, ReA is classified as a type of seronegative spondyloarthropathy [13]. Although post-infection arthritis has long been documented, the term “reactive arthritis” was not introduced until 1969 by Ahvonen and colleagues to describe arthritis that occurs soon after or during an infection elsewhere in the body, with no evidence of microbial entry into the joint [14, 15]. For a considerable period, ReA was considered as a sequel to infection. It is widely accepted that ReA is caused by pathogenic microbial infections, host genetic and immune factors, and environmental factors.

### Pathogenic microorganisms

Common pathogens responsible for ReA include *Chlamydia trachomatis*, which infects the genitourinary system, and *Yersinia, Salmonella, Shigella*, or *Campylobacter*, which infect the digestive system [16, 17]. The fundamental characteristic of reactive arthritis lies in continuous stimulation or “reaction” of the immune system to an ongoing or cleared infection, resulting in intermittent exacerbations of various immune-mediated signs and symptoms [18]. In principle, ReA can be caused by any pathogenic microbial infection. For example, post-streptococcal ReA [19, 20], parasitically induced ReA [21–23], and COVID-19-related ReA have been reported [24–26]. From a broader perspective, even non-pathogenic microbial infections can serve as triggering factors for ReA. Any stimulus capable of causing excessive stress on the body has the potential to induce reactive arthritis; examples include adjuvant-induced arthritis [27] and post-vaccination reactive arthritis [28].

### Host aspects

Numerous studies have demonstrated that a positive HLA-B27 status is a predisposing factor for ReA. Reports indicate that individuals who are B27-positive exhibit a 5–10 times higher prevalence of developing ReA compared to the general population [29]. Moreover, the prevalence among B27-positive relatives of such patients is tenfold greater than average [30]. Additionally, the incidence rate of ReA is influenced by age and gender. The interaction between the host immune system and pathogenic microorganisms is the immediate cause of ReA, which will be further discussed in the subsequent section.

### Environmental factors

Numerous environmental risk factors have been identified for SpA, particularly for axial spondyloarthritis (AxSpA), ReA, and psoriatic arthritis (PsA) [31]. Smoking, dietary changes, and obesity are associated with the development and severity of SpA. Research has shown that the frequency and severity of ReA in Canada may be linked to foodborne illnesses, water quality, and treatment of sexually transmitted infections [32].

## Role of the immune system in the pathogenesis of reactive arthritis

The perplexing question remains as to how an infection outside the joint can trigger an inflammatory response within it. The host immune system may contribute to the development of ReA through various mechanisms:

1. Homing theory: pathogens enter mononuclear phagocytes from mucous membranes in either the genitourinary or gastrointestinal tract before reaching joints via blood circulation. Bacterial degradation products such as lipopolysaccharide (LPS), heat-shock protein 60 (Hsp60), DNA/RNA [33–35], and even viable *Chlamydia* organisms have been detected in synovial fluid from affected joints in ReA patients [36]. Experiments in mice have demonstrated that P‑selectin expression on endothelial cells can be induced by monocytes with intracellular bacterial components, which may lead microbial antigens into previously healthy joints [37, 38]. Microbial products like LPS, peptidoglycan, zymosan, or bacterial outer membranes act as TLR ligands contributing to articular TLR expression induction resulting in joint inflammation [39–41].
2. Molecular mimicry theory: HLA-B27 binds to a unique peptide derived from microbes presenting it to CD8-positive T cells; alternatively, some microbial antigen structures are similar to HLA-B27, or HLA-B27 itself may be the target of the immune response [42]. Ultimately, this is recognized by CD8+ T cells [43], resulting in cross-reactivity and an HLA–B27–restricted cytotoxic T cell response [44]. It is suggested that this then leads to autoimmunity and inflammation with tissue damage.
3. Cytokine imbalance theory: for example, T‑helper 1 cytokines, such as tumor necrosis factor alpha (TNF-α) and interferon-gamma (IFNγ), are crucial for the effective elimination of bacteria, whereas T‑helper 2 cytokines, such as interleukin (IL)-4, or T‑helper 3 cytokines, such as IL-10, may inhibit effective elimination [43]. A “wrong” cytokine pattern contributes to the pathogen not being effectively cleared [16, 43], resulting in persistent immune inflammation forming chronic arthritis. The synovial inflammation is followed by erosion, exposition of cartilage antigens, and autoimmunity [45], which further aggravates the inflammatory response.
4. Immune complex theory: microbial antigens may also appear in the circulation, perhaps as part of the immune complexes (ICs) [46]. It has been demonstrated that ICs consisting of *Yersinia* antigen and specific antibodies are found in the circulation as well as in synovial fluid in *Yersinia*-triggered ReA [47]. The specific antibodies together with microbial antigens in the joint may participate in inflammation and tissue injury through complement activation [48].

**Fig. 1:**
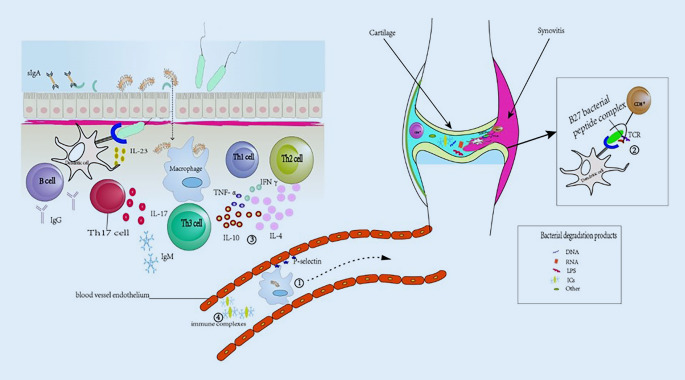
Reactive arthritis may be mediated by the immune system through various mechanisms: ① The pathogen enters the joint from the primary site with mononuclear phagocytes as the carrier, and the degradation products of the pathogen are used as antigens to induce immune response. ② HlA-B27 binds with antigenic peptides from pathogens for recognition by autoreactive CD8+ T cell receptors (*TCR*). ③ Cytokine imbalances cause inefficient clearance of pathogens, resulting in sustained immune responses. ④ Microbial antigens may also form immune complexes with specific antibodies, which are deposited in the joints through the blood circulation and activate the immune system

Figure 1 shows the mechanism of action of the immune system in reactive arthritis.

**Fig. 2:**
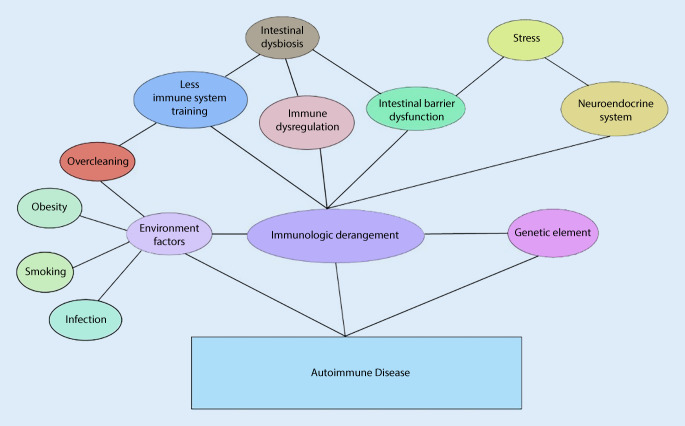
Overview of the pathogenesis of autoimmune diseases. Intestinal microecological disorders and stress contribute to contribute to impaired intestinal barrier function, disrupted immune regulation balance, compromised normal immune response training, and neuroendocrine system disturbances, ultimately leading to immune dysfunction; meanwhile combined effects of environmental and genetic factors eventually culminate in autoimmune diseases. The process of autoimmune diseases involves complex multifactor interactions, which cannot be fully enumerated here

## Immune, inflammatory, and autoimmune diseases

Although these mechanisms may explain how pathogenic microorganisms induce the host immune system to mediate arthritis, many questions remain unanswered. Why do only 1–10% of people develop reactive arthritis after infection [49]? The natural course of ReA is usually 4–5 months [50]. Why do some patients develop chronic arthritis? ReA is a multisystem autoimmune disease. What is the pathogenesis underlying extra-articular lesions? One hypothesis is that the acute antibacterial response is followed by a self-perpetuating autoimmune response against an autologous peptide [51]. From our perspective, these immune-mediated pathological processes can be summarized as an exaggerated response to pathogenic microbial infection on a macroscopic level. The overactivation of the immune system leads to hyperfunction of the body, resulting in multisystem immune damage throughout the body, including arthritis.

Our immune system is composed of immune organs, cells, and molecules. It is well known that the immune system has three main functions: 1) detecting and eliminating foreign bodies and pathogenic microorganisms; 2) monitoring and removing mutated tumor cells, senescent cells, dead cells, or other harmful components within the body; c) maintaining a stable internal environment through autoimmune tolerance and regulation.

Inflammation is a defensive response to injurious stimuli through a complex set of interactions among soluble factors and cells [52]. When the traumatic stimulation occurs, inflammatory mediators are released, leading to increased vascular permeability while activating the immune system, resulting in complex inflammatory responses that resist invasion by pathogens while promoting tissue repair. We face challenges from pathogenic microorganisms daily, with our internal environment’s stability maintained by our immune system playing an essential role.

In general, both immune and inflammatory responses are beneficial. However, if there is an imbalance in the immune system, the inflammatory response is out of control, or the immune system does not properly recognize self and non-self and wrongly attacks the components of the body, it will lead to damage of tissues and organs. Current research has shown that intestinal microbial ecological disorders [53–55], along with stress disorders, play significant roles in autoimmune diseases [56–58].

Physical and psychological stresses have been implicated in the development of autoimmune disease, as evidenced by numerous animal and human studies demonstrating their impact on immune function [57, 59]. Abnormalities in the microbial community, particularly within the intestinal symbiosis, have also been observed in patients with autoimmune diseases [60]. The microbiome plays a crucial role in host immune system development and immune regulation. Interventions that target the microbiome are emerging as new therapeutic strategies to prevent or cure autoimmune diseases [50]. The following sections will discuss how microbiological and stress disorders affect our immune system based on the current research results, to provide new ideas for the prevention and treatment of autoimmune diseases such as reactive arthritis.

## Microbes shape the immune system

Our bodies are colonized by more than a hundred trillion commensals, represented by viruses, bacteria, and fungi [61, 62]. Microbes are widely distributed in our skin, gastrointestinal tract, respiratory tract, and female vaginal mucosal surface. When we are born, we are first exposed to the microbes in our mother’s vagina or the air, and then the microbes begin to settle on our bodies and stay with us for life. Intriguingly, it has been pointed out that when we are still in utero, we begin to interact with microbes [63–65].

More than 100 trillion microorganisms, mostly bacteria, colonize our gastrointestinal tract. Over millions of years of coevolution, a balanced and mutually beneficial state has been achieved between the host and the microbe [66, 67]. The gut microbiota interacts with and regulates the host immune system in various ways.

### Compose and regulate the gastrointestinal barrier system

The intestinal mucosal barrier, which is composed of epithelial cells and mucus layers secreted by goblet cells and contains commensal bacteria, constitutes the first line of defense against pathogenic gut microbiota [68]. The microbiota is essential in the defense against pathogens once they compete for nutrients and adhesion sites, some even actively eliminating competition by secreting antimicrobial peptides [69]. The mucus layer can effectively prevent the adhesion of pathogenic microorganisms to the intestinal mucosa, and it is also the site of specific secretory immunoglobulin A(S-IgA) produced by intestinal-associated lymphoid tissue (GALT). The mechanical and biological barrier composed of intestinal mucosal epithelial cells, close connections between cells, and symbiotic bacteria can prevent pathogenic microorganisms, toxic substances, and food antigens from entering the blood circulation through the intestinal mucosa. Disruption of gut dysbiosis or barrier function can trigger loss of tolerance towards gut microbial antigens, eliciting immune responses that can potentially promote not only local inflammation but also distal autoimmune phenomena [70]. In ReA originating from an intestinal infection scenario, components of pathogens in joints likely enter the bloodstream through damaged intestinal barriers before reaching joints. This seems to be supported by a large number of studies showing a close relationship between inflammatory bowel disease and spondylarthritis [71–73].

### Microbes induce and regulate the immune system

Contrary to our intuition, gut microbiota does not simply evade the immune system to persist in the intestinal tract. There is a complex interplay between the local microbiome, the intestinal epithelium, and resident immune cells that actively foster gastrointestinal homeostasis [74]. The constitutive sensing of local microbial products plays an intrinsic role in maintaining epithelial integrity and barrier function [75]. For instance, certain components of the gut microbiota have the ability to regulate immune cell function within the gut. In animal studies, when purified *Bacteroides fragilis* polysaccharide A (PSA) is administered to animals, it can suppress proinflammatory interleukin-17 production by intestinal immune cells and also inhibit reactions in cell cultures conducted in vitro [76]. Furthermore, PSA provides protection against inflammatory diseases through its functional requirement for interleukin-10-producing CD4+ T cells [76].

Compelling evidence from genetics studies, translational research, and animal models implicates the IL-23/IL-17 axis and related cytokines in the pathogenesis of SpA [77]. Consumption of *L. casei* abrogated the expression of TNF‑α, IL-17, IL-23, IL-1β, and IL‑6 in cecum and mesenteric lymph nodes of mice, and these cytokines are needed for differentiation of immune cells involved in the development of reactive arthritis such as Th17 and γδ T cells [77].

Regulatory T cells (Tregs) are a crucial subset of CD4+ T cells that play a pivotal role in immune regulation. Treg cells (CD4 + CD25 + Foxp3) exhibit high IL-10 production and are under the control of the Foxp3transcription factor [78]. They are responsible for regulating intestinal inflammation and maintaining immune homeostasis [79, 80]. Accumulating evidence suggests that the presence of the gut microbiota, particularly the *Clostridium *species, affects the development and function of Treg cells and that these bacteria-induced Treg cells in the intestine likely contribute to tolerance towards gut microbiota [81–83]. In cases where the gut immune system loses its ability to regulate the microbiome, immune activation may lead to abnormal migration of intestinal macrophages and lymphocytes from inflamed gut mucosa to joints or other tissues affected by spondylarthritis [75].

### Immunomodulatory effects of microbial metabolites

The intestinal mucosa is constantly exposed to various external antigens such as food antigens, food-borne pathogens, and commensal microbes residing in the intestinal lumen [60]. The gastrointestinal (GI) tract’s immune system faces continuous antigenic challenges from the lumen and must therefore effectively distinguish between tolerable and non-tolerable antigens [69]. Intestinal microbiota produces diverse metabolites through decomposition, modification, synthesis, and other functions to regulate host immunity and ensure their survival while maintaining intestinal barrier function and providing nutrients to the host. Intestinal microorganisms produce a series of metabolites through decomposition, modification, synthesis, and other functions to regulate host immunity, to maintain their survival while maintaining intestinal barrier function and providing nutrients to the host. The diversity of metabolites produced by gut microbiota and their mechanisms of action remain largely unclear. This article highlights only a few common metabolites and their effects on the host immune system.

Short-chain fatty acids (SCFAs), including acetic acid, butyric acid, and propionic acid, are major products of anaerobic bacterial fermentation in the intestine [84]. In addition to their important role as fuel for intestinal epithelial cells, SCFAs have broad effects on the host immune system [84, 85]. SCFAs inhibit histone deacetylases (HDACs), and this tends to promote a tolerogenic, anti-inflammatory cell phenotype that is crucial for maintaining immune homeostasis [86]. Animal studies have shown that SCFAs can regulate the size and function of the colonic Treg pool and protect against colitis in a Ffar2 (GPR43)-dependent manner in mice [80].

Taurine is a bile acid component modulated by commensal bacteria. Maayan Levy et al. identified the organic acid taurine as a mucosal inflammasome activator and the metabolites histamine and spermine as inflammasome inhibitors [87]. Microbial metabolites signal to the NLRP6 inflammasome, thereby activating host immune circuits to orchestrate an antimicrobial program, which optimizes commensal colonization and persistence [87].

Dietary tryptophan is processed into several different indole derivatives by the bacteria of the gut microbiome, which can act as ligands for the aryl hydrocarbon receptor (AhR) in host cells [63]. The AhR is a ligand-activated transcription factor with a central role in the intestinal mucosa [63]. In summary, metabolites derived from gut microbiota contribute to the proper function of the epithelial barrier regulate host immune function, and maintain immune homeostasis through various pathways.

### Immune training of microbiota

In industrialized countries, there has been a significant increase in the prevalence of allergic and autoimmune diseases over the past few decades, while major infectious and parasitic diseases have shown a decline [88, 89]. The hygiene hypothesis, despite being controversial, sheds light on the mechanisms underlying various immune-related diseases, particularly autoimmune disorders [90]. Over the past three decades, the hygiene hypothesis has been supported by concepts and findings coming from a variety of scientific disciplines such as epidemiology, immunology, microbiology, and anthropology [91].

Although our understanding of how microbes influence the immune system is incomplete, it is evident that they play a role in training our immune response. Recent discoveries indicate that not only adaptive immunity but also innate immunity can be influenced by previous exposure to pathogens or their products, thus mounting resistance to reinfection, a phenomenon termed trained immunity [92]. Through continuous trial and error with corrective measures, our immune function maintains an optimal balance between being too weak or excessively reactive. When operating optimally, the interplay between the immune system–microbiota alliance integrates both innate and adaptive arms of immunity to select, calibrate, and terminate responses appropriately [93]. Excessive cleanliness in our environment has resulted in a reduced requirement for infectious stimuli necessary for the proper development of the immune system [94], similar to how an inadequately trained army would either be ineffective or overly vigilant towards every potential threat.

## Stress and immunologic derangement

As the medical model shifts from traditional biomedical to biopsychosocial factors, these are increasingly recognized as playing a prominent role in autoimmune diseases. From a biological perspective, stress is not inherently negative. In fact, in response to danger, the stress response can enhance organ function and activate an individual’s adaptive capacities to environmental challenges. However, long-term chronic stress or extreme stress, beyond the body’s capacity for adaptation, will cause the occurrence of related diseases. It is currently believed that stress causes autoimmune diseases via two main mechanisms: 1) direct action: stress-triggered neuroendocrine hormones lead to immunologic derangement, which ultimately results in autoimmune disease by altering or amplifying cytokine production [56]; 2) indirect action: stress has a detrimental effect on the intestinal barrier, which in turn affects the immune system and ultimately leads to disruption of immune homeostasis [58].

Glucocorticoids and catecholamines are released by the hypothalamic–pituitary–adrenal axis during stress [95]. Adrenergic receptors are present on cell membranes throughout the innate and adaptive arms of the immune system, including B and T lymphocytes, macrophages, monocytes, and natural killer cells [96]. By downregulating type 1 and upregulating type 2 cytokine secretion, both glucocorticoids and catecholamines may cause selective suppression of cellular immunity and a shift toward Th2-mediated humoral immunity [97]. Stress hormones, through modulation of the systemic or local pro-/anti-inflammatory and Th1/Th2 cytokine balance [98], may affect the susceptibility to or the course of autoimmune and atopic/allergic diseases [99].

Psychological stress has been shown to influence the clinical course of chronic intestinal disorders such as inflammatory bowel disease [100]. Animal models also demonstrate that different types of psychological and physical stress induce dysfunction in the intestinal barrier, leading to increased uptake of potentially harmful substances (e.g., antigens, toxins, and other proinflammatory molecules) from the gut lumen [101–103]. For example, rat offspring exposed to prolonged maternal separation during their stress hyporesponsive period become predisposed to developing several adverse health effects [104]. First and foremost, among these is an increased vulnerability towards developing heightened intestinal permeability in response to a chronic psychological stressor [104].

## Conclusion

Previous studies on ReA have primarily focused on joint symptoms following infection by specific pathogenic microorganisms but rarely explored ReA’s pathogenesis originating from host factors. In reality, ReA shares similarities with autoimmune diseases like ankylosing spondylitis and rheumatoid arthritis. Autoimmunity is a case of mistaken identity, where the immune system mistakenly targets self-tissues and cells as if they were pathogens [105]. Clinical studies and animal experiments have demonstrated that autoimmune diseases are closely associated with genetic, immune, and environmental factors. Because genetic factors are beyond our control, studies on the interaction between environment and immunity become more meaningful.

Although it cannot be denied that ReA caused by pathogens such as *Chlamydia trachomatis *or *Yersinia* is relatively common, an increasing number of reports have confirmed that ReA is not solely triggered by urinary tract or gastrointestinal infections. Microbial infection at most serves as an inducing factor, while immune dysfunction resulting from factors like microecological imbalance or stress state constitutes the internal basis for ReA development, which can also apply to other autoimmune diseases. The effects of intestinal microecology and stress on the immune system have attracted the attention of more and more researchers. Fecal microbiota transplantation is currently being investigated in clinical trials for treating various autoimmune diseases [106]. Although there remain numerous problems to be explored in this field and current technology cannot objectively assess an individual’s immune status, we believe that with technological advancements and extensive research efforts, new prospects will emerge for preventing and treating autoimmune diseases typified by ReA.

## Data Availability

All data and materials are authentic and usable.

## Abbreviations

AhR: Aryl hydrocarbon receptor
AxSpA: Axial spondyloarthritis
ICs: Immune complexes
IFNγ: Interferon gamma
LPS: Lipopolysaccharide
PSA: Polysaccharide A
PsA: Psoriatic arthritis
ReA: Reactive arthritis
SCFAs: Short-chain fatty acids
TLR: Toll-like receptors
TNF‑α: Tumor necrosis factor alpha
Tregs: Regulatory T cells

## Declarations

For this article no studies with human participants or animals were performed by any of the authors. All studies mentioned were in accordance with the ethical standards indicated in each case.
